# Serum Cytokines and Growth Factors in Subjects with Type 1 Diabetes: Associations with Time in Ranges and Glucose Variability

**DOI:** 10.3390/biomedicines11102843

**Published:** 2023-10-19

**Authors:** Vadim V. Klimontov, Kamilla R. Mavlianova, Nikolai B. Orlov, Julia F. Semenova, Anton I. Korbut

**Affiliations:** 1 Laboratory of Endocrinology, Research Institute of Clinical and Experimental Lymphology—Branch of the Institute of Cytology and Genetics, Siberian Branch of Russian Academy of Sciences (RICEL—Branch of IC&G SB RAS), 630060 Novosibirsk, Russia; 2 Laboratory of Clinical Immunogenetics, Research Institute of Clinical and Experimental Lymphology—Branch of the Institute of Cytology and Genetics, Siberian Branch of Russian Academy of Sciences (RICEL—Branch of IC&G SB RAS), 630060 Novosibirsk, Russia

**Keywords:** type 1 diabetes, inflammation, cytokines, growth factors, hyperglycemia, time in range, glucose variability, continuous glucose monitoring

## Abstract

The detrimental effect of hyperglycemia and glucose variability (GV) on target organs in diabetes can be implemented through a wide network of regulatory peptides. In this study, we assessed a broad panel of serum cytokines and growth factors in subjects with type 1 diabetes (T1D) and estimated associations between concentrations of these molecules with time in ranges (TIRs) and GV. One hundred and thirty subjects with T1D and twenty-seven individuals with normal glucose tolerance (control) were included. Serum levels of 44 cytokines and growth factors were measured using a multiplex bead array assay. TIRs and GV parameters were derived from continuous glucose monitoring. Subjects with T1D compared to control demonstrated an increase in concentrations of IL-1β, IL-1Ra, IL-2Rα, IL-3, IL-6, IL-7, IL-12 p40, IL-16, IL-17A, LIF, M-CSF, IFN-α2, IFN-γ, MCP-1, MCP-3, and TNF-α. Patients with TIR ≤ 70% had higher levels of IL-1α, IL-1β, IL-6, IL-12 p70, IL-16, LIF, M-CSF, MCP-1, MCP-3, RANTES, TNF-α, TNF-β, and b-NGF, and lower levels of IL-1α, IL-4, IL-10, GM-CSF, and MIF than those with TIR > 70%. Serum IL-1β, IL-10, IL-12 p70, MCP-1, MCP-3, RANTES, SCF, and TNF-α correlated with TIR and time above range. IL-1β, IL-8, IL-10, IL-12 p70, MCP-1, RANTES, MIF, and SDF-1α were related to at least one amplitude-dependent GV metric. In logistic regression models, IL-1β, IL-4, IL-10, IL-12 p70, GM-CSF, HGF, MCP-3, and TNF-α were associated with TIR ≤ 70%, and MIF and PDGF-BB demonstrated associations with coefficient of variation values ≥ 36%. These results provide further insight into the pathophysiological effects of hyperglycemia and GV in people with diabetes.

## 1. Introduction

The burden of type 1 diabetes (T1D) is enormous and is expected to increase. In 2021, there were about 8.4 million individuals worldwide living with T1D. The remaining life expectancy of a 10-year-old diagnosed with T1D ranges from a mean of 13 years in low-income countries to 65 years in high-income ones [1]. Vascular diabetes complications remain an important determinant of mortality in subjects with T1D [2,3,4]. The Diabetes Control and Complications Trial (DCCT) and its longitudinal observational follow-up study, the Epidemiology of Diabetes Interventions and Complications (EDIC) study, clearly showed a reduction in the incidence of diabetic retinopathy, diabetic nephropathy, diabetic neuropathy, and cardiovascular disease (CVD) outcomes, following the optimization of glycemic control in people with T1D [5,6]. However, the benefits of intensive insulin treatment in terms of the risk of major adverse cardiovascular events vary substantially between individuals with T1D [7]. Age, diabetes duration, and glucose level do not completely explain the associations between complications and mortality [4]. This underlines the necessity for a better understanding of the drivers of diabetic complications.

Clinicians noticed a long time ago that many patients with excessive glucose fluctuations and unstable glycemic control rapidly develop complications. One of the first experimental studies testing the deteriorating effect of glucose fluctuations on the kidneys was published in 1957: it showed the rapid development of glomerulosclerosis in rats when excessive blood glucose fluctuations were induced by the intermittent administration of glucose and insulin [8]. In recent years, the role of glucose variability (GV) as a trigger of diabetic vascular complications has attracted increasing attention [9,10]. In the observational Finnish Diabetic Nephropathy (FinnDiane) study, glycated hemoglobin A1c (HbA1c) variability predicts incident cardiovascular events, microalbuminuria, and overt diabetic nephropathy in subjects with T1D [11]. A recent analysis of 14 studies with 254,017 patients with diabetes revealed that the highest HbA1c variability is associated with increased risks of CVD. The CVD risk associated with HbA1c variability might be even higher among patients with T1D [12]. However, the role of short-term GV as a trigger of diabetic complications remains to be clarified. 

Continuous glucose monitoring (CGM) opened the way for the comprehensive assessment of short-term GV in patients with diabetes. Two principal dimensions of GV, amplitude and time, can be assessed and visualized from CGM data [9]. Time in range (TIR), time above range (TAR), and time below range (TBR) became standardized CGM metrics for clinical care [13]. Accumulating evidence suggests a negative association between time in range (TIR) and microvascular complications in subjects with T1D [14,15]. In addition, associations of coefficient of variation (CV) and the mean amplitude of glycemic excursions (MAGE) with diabetic microvascular complications [16] and cardiovascular autonomic neuropathy [17] were reported. 

The mechanisms of the harmful effects of excessive GV on target organs in diabetes have been intensively studied in recent years. The cardiovascular system, pancreas, adipose and muscle tissues, gastrointestinal tract, and kidney have been recognized as the loci with the highest expression of GV-related genes [18]. Current data indicate that GV effects can be realized through oxidative stress, non-enzymatic glycation, chronic low-grade inflammation, endothelial dysfunction, platelet activation, impaired angiogenesis, and renal fibrosis. At the molecular level, these pathophysiological processes may be mediated through shifts in the production of cytokines and growth factors playing an important role in intercellular interactions [19]. At present, the relationships between GV and changes in the production of these regulators are not well understood. 

The large number of secreted cytokines and their interactions make it difficult to assess changes in the cytokine response during stress and disease. Moreover, measuring individual regulators can lead to a one-sided or even incorrect interpretation of the response. In such situations, multiplex platforms, widely used to measure multiple biomarkers from a single assay, may have advantages over single assay-based detection methods [20,21]. In recent years, a multiplex bead assay has become increasingly popular in studies of cytokine panels in various diseases, including diabetes [22,23,24]. 

Therefore, we aimed to assess a broad panel of circulating cytokines and growth factors in subjects with T1D via a multiplex bead array assay and to determine the associations of these regulators with CGM-derived TIR and GV parameters.

## 2. Materials and Methods

### 2.1. Design

We performed a cross-sectional observational single-center comparative study. 

Caucasian male and female patients with T1D aged 18 to 70 years were included. Acute infections within three months prior to the study, pregnancy, malignant neoplasm, chronic inflammatory or autoimmune diseases, current diabetic ketoacidosis or hyperglycemic hyperosmolar state, end-stage renal disease, and diabetic foot syndrome were established as the principal exclusion criteria. Subjects without the above-mentioned diseases and conditions who had normal glucose tolerance (NGT) verified by the results of the oral glucose tolerance test and HbA1c measurement were included in the control group.

All study participants underwent a detailed clinical examination with real-time CGM. Digital CGM data were used for the calculation of TIRs and GV parameters. The panel of serum cytokines and growth factors was assessed with a multiplex bead array assay.

**Figure 1:**
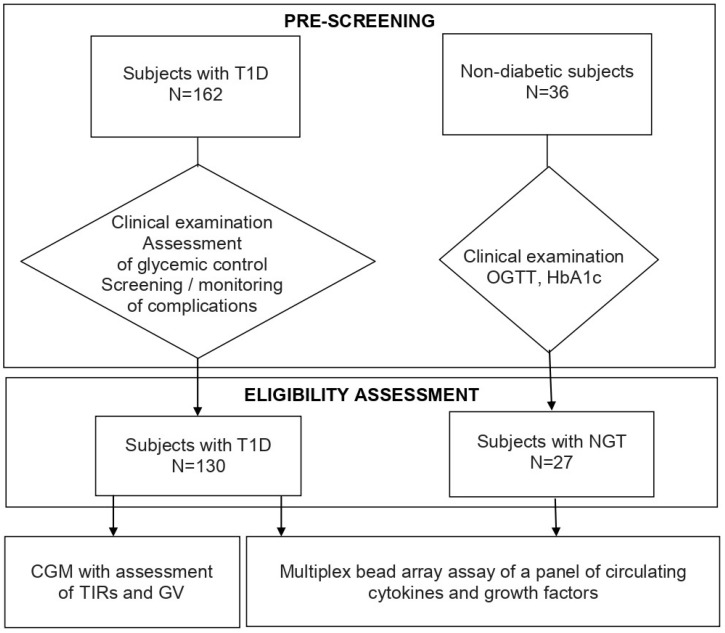
Study design. CGM, continuous glucose monitoring; GV, glucose variability; HbA1c, glycated hemoglobin A1c; NGT, normal glucose tolerance; OGTT, oral glucose tolerance test; T1D, type 1 diabetes; TIRs, time in ranges.

The study design is shown in Figure 1.

### 2.2. Methods

General clinical tests. The levels of HbA1c, serum biochemical parameters, and urinary albumin were assessed in patients with T1D with the use of a AU480 Chemical Analyzer (Beckman Coulter, Brea, CA, USA) and commercially available cartridges. A complete blood count was performed on a hematology analyzer (Analyticon Biotechnologies AG, Lichtenfels, Germany). The fasting C-peptide was determined using chemiluminescent immunoassay with an Immulite 2000 XPi immunological analyzer (Siemens Healthineers, Erlangen, Germany). 

A standard oral glucose tolerance test with 75 g of glucose was performed in non-diabetic subjects. Blood samples for the measurement of glucose were taken from the cubital vein at 0 and 120 min of the test.

CGM parameters. Patients with T1D underwent real-time CGM within 3–13 days (median 5.4 days) with MMT-722 or MMT-754 monitoring systems and CareLink^®^ Pro software 2.5.524.0 (version 2.5A, Medtronic, Minneapolis, MN, USA). Study participants were instructed to calibrate the system at least three times a day. The results of the CGM of each subject were reviewed individually and recording defects were eliminated. 

TIRs were defined according to the International Consensus on Use of Continuous Glucose Monitoring [25] and the International Consensus on Time in Range [13]. Specifically, we assessed TIR: 70–180 mg/dL (3.9–10.0 mmol/L), time above range, level 1 (TAR L-1: 181–250 mg/dL [10.1–13.9 mmol/L]), time above range, level 2 (TAR L-2: >250 mg/dL [>13.9 mmol/L]), time below range, level 1 (TBR L-1: 54–69 mg/dL [3.0–3.8 mmol/L]), and time below range, level 2 (TBR L-2: <54 mg/dL [<3.0 mmol/L]). In addition, the following GV parameters were calculated: CV, MAGE, and mean absolute glucose (MAG) changes. Among these parameters, CV is an indicator of variations around the mean glucose, MAGE is the mean differences from peaks to nadirs, and MAG is the absolute differences between sequential readings divided by the time [9,10]. The GV parameters were estimated with the use of EasyGV v. 9.0.R2 software [26]. The values of TIR > 70% were considered as targets [13] and values of CV < 36% as indicators of stable glucose level [25].

Serum cytokines and growth factors. Serum samples for the assay of cytokines and growth factors were obtained from the fasting blood and stored at −80 °C until the analysis. Repeated freeze–thaw cycles were avoided. 

The concentrations of interleukin 1 alpha (IL-1α), interleukin 1 beta (IL-1β), interleukin-1 receptor antagonist (IL-1Ra), interleukin 2 (IL-2), interleukin-2 receptor alpha chain (IL-2Rα), interleukin 3 (IL-3), interleukin 4 (IL-4), interleukin 5 (IL-5), interleukin 6 (IL-6), interleukin 7 (IL-7), interleukin 8 (IL-8), interleukin 9 (IL-9), interleukin 10 (IL-10), interleukin-12 subunit beta (IL-12 p40), interleukin 12 active heterodimer (IL-12 p70), interleukin 16 (IL-16), interleukin 17A (IL-17A), interleukin 18 (IL-18), leukemia inhibitory factor (LIF), granulocyte colony-stimulating factor (G-CSF), granulocyte-macrophage colony-stimulating factor (GM-CSF), macrophage colony-stimulating factor (M-CSF), chemokine growth-regulated protein alpha (GRO-α), interferon alpha 2 (IFN-α2), interferon gamma (IFN-γ), interferon gamma-induced protein 10 (IP-10), monocyte chemoattractant protein 1 (MCP-1), monocyte chemoattractant protein 3 (MCP-3), macrophage migration inhibitory factor (MIF), monokine induced by interferon-γ (MIG), macrophage inflammatory protein 1 alpha (MIP-1α), macrophage inflammatory protein 1 beta (MIP-1β), regulated on activation, normal T cell expressed and secreted (RANTES), tumor necrosis factor alpha (TNF-α), tumor necrosis factor-beta (TNF-β), TNF-related apoptosis-inducing ligand (TRAIL), soluble cytotoxic factor (SCF), stem cell growth factor beta (SCGF beta), stromal cell-derived factor-1 alpha (SDF-1α), basic fibroblast growth factor (bFGF), platelet-derived growth factor subunit B (PDGF-BB), hepatocyte growth factor (HGF), beta subunit of nerve growth factor (β-NGF), and vascular endothelial growth factor (VEGF) were assessed with the use of Bio-Plex Pro™ Human Cytokine Screening Panel Assay (Bio-Rad Laboratories, Hercules, CA, USA).

A multiplex bead array assay was performed according to the manufacturer’s instructions. Serum samples were centrifuged at 10,000× *g* for 10 min at 4 °C, diluted 1:4 with Bio-Plex Sample Diluent, and incubated with antibody-coupled beads, detection antibody, and streptavidin for 30 min, 30 min, and 10 min, respectively. After each coating with antigen, the plate was washed with a Bio-Plex Handheld Magnetic Washer, resuspended, and vortexed. Fluorescence was measured on a two-beam laser automated analyzer Bio-Plex^®^ 200 system. Data were acquired with Bio-Plex Manager Software 4.0. The values below the detection limit were set to zero.

### 2.3. Statistical Analysis

Statistics 12.0 software package (Dell, Round Rock, TX, USA) was used for analysis. The outliers were excluded with Dixon’s Q test. Quantitative data are presented as medians (lower quartiles; upper quartiles); frequencies are presented as percentages (%). The Kolmogorov–Smirnov (KS) test was applied to test the normality of data distribution. As most of the studied parameters were not distributed normally, a non-parametric Mann–Whitney U-test was used for group comparisons. Spearman’s rank correlation analysis and logistic regression analysis were applied to test the associations between studied parameters. *p*-values less than 0.05 were considered significant.

## 3. Results

### 3.1. Clinical Characteristics of the Study Participants

One hundred and thirty subjects with T1D, fifty-five men and seventy-five women, aged from 18 to 70 years (median 33 years), with diabetes duration from 0.5 to 55 years (median 15 years), were included in the study. Eighty eight patients had normal body mass index (BMI), twenty-four were overweight, and eighteen individuals had obesity. The level of fasting C-peptide in most patients was below the sensitivity limit (<0.1 ng/mL); however, 19 subjects had detectable C-peptide (range: 0.102–1.3 ng/mL). Eighty-two individuals received multiple daily injections of insulin and forty-eight were on continuous subcutaneous insulin infusion. Clinical characteristics are presented in Table 1.

Twenty-seven non-obese individuals with NGT, twelve men and fifteen women, from 19 to 62 years of age (median 32 years), were included in the control group.

Mean monitoring glucose, CGM-derived TIRs, and GV parameters in subjects with T1D are presented in Table 2.

### 3.2. Serum Cytokines and Growth Factors in Subjects with NGT and T1D

**Figure 2:**
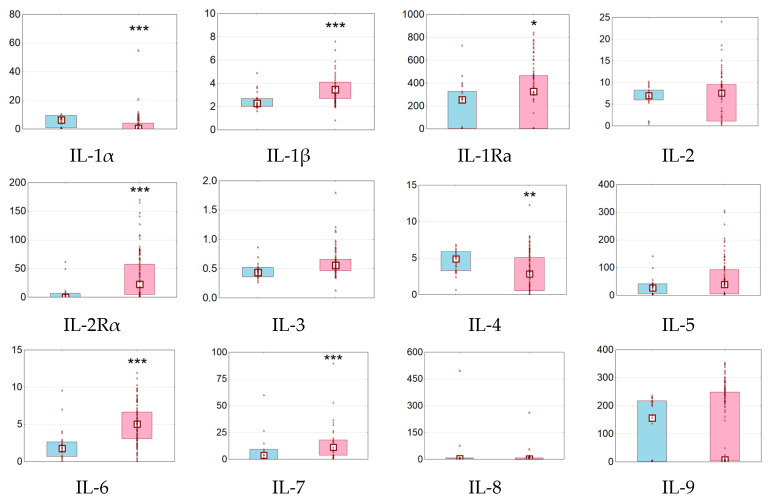

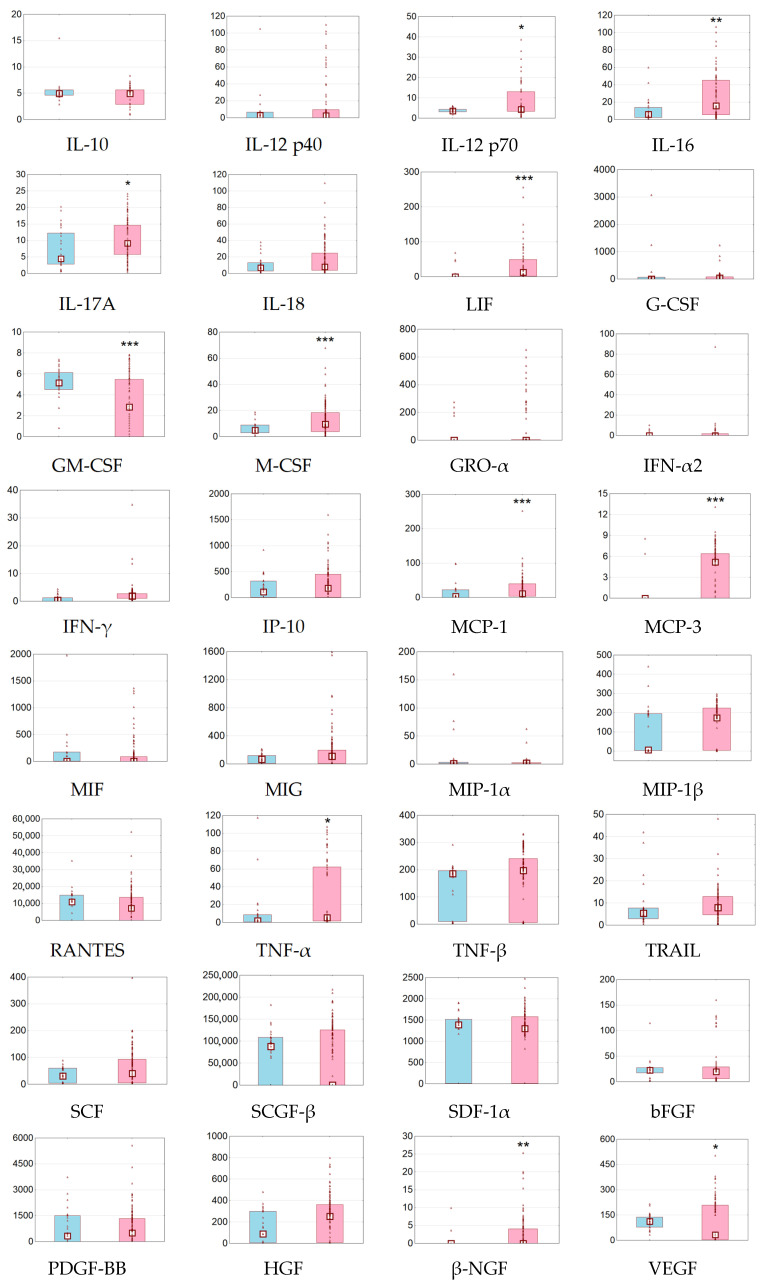
Serum concentrations of cytokines and growth factors in subjects with NGT and T1D. Data are presented as raw data (dots), medians (squares), and interquartile ranges (boxes). NGT subjects are marked in blue and patients with T1D are marked in pink. The concentrations are expressed in pg/mL. * *p* < 0.05, ** *p* < 0.01, *** *p* < 0.001 vs. subjects with NGT. NGT, normal glucose tolerance; T1D, type 1 diabetes.

Patients with T1D as compared to subjects with NGT demonstrated significant increases in serum levels of IL-1β, IL-1Ra, IL-2Rα, IL-3, IL-6, IL-7, IL-12 p70, IL-16, IL-17A, LIF, M-CSF, IFN-α2, IFN-γ, MCP-1, MCP-3, and TNF-α (Figure 2).

In addition, there were trends towards an increase in the levels of G-CSF, SCF, TRAIL, and HGF. On the other hand, concentrations of IL-1α, IL-4, and GM-CSF were decreased and MIF demonstrated a tendency to decrease. Other molecules showed no significant changes (Figure 2).

### 3.3. Serum Cytokines and Growth Factors in Subjects with T1D: Relationships with TIRs

The changes in the levels of the most studied molecules were more pronounced in subjects with TIR < 70%; in this group we found significant increases in the levels of IL-1β, IL-1Ra, IL-2Rα, IL-3, IL-6, IL-7, IL-12 p40, IL-16, IL-17A, G-CSF, M-CSF, IFN-γ, MCP-1, MCP-3, TNF-α, TNF-β, SCF, and HGF, and decreases in the levels of IL-1α, IL-4, IL-8, IL-10, GM-CSF, and MIF when compared to control (Table 3). In patients with TIR > 70%, concentrations of IL-1α, IL-1β, IL-2Rα, IL-3, IL-4, IL-7, IL-16, IL-17A, LIF, G-CSF, IFN-α2, IFN-γ, MCP-1, MCP-3, and β-NGF were significantly different from control.

Among subjects with diabetes, those with TIR ≤ 70% had higher levels of IL-1α, IL-1β, IL-6, IL-12 p70, IL-16, LIF, M-CSF, MCP-1, MCP-3, RANTES, TNF-α, TNF-β, and b-NGF, and lower levels of IL-1α, IL-4, IL-10, GM-CSF, and MIF than individuals with TIR > 70% (Table 3).

We found weak correlations between TIR and concentrations of IL-1β (r = −0.21, *p* = 0.02), IL-10 (r = 0.23, *p* = 0.008), IL-12 p70 (r = −0.26, *p* = 0.004), MCP-1 (r = −0.32, *p* = 0.0002), MCP-3 (r = −0.19, *p* = 0.03), RANTES (r = −0.24, *p* = 0.006), SCF (r = −0.18, *p* = 0.04), and TNF-α (r = −0.23, *p* = 0.008). Most of these cytokines demonstrated correlations with TAR L1: IL-1β (r = 0.23, *p* = 0.008), IL-10 (r = −0.21, *p* = 0.02), IL-12 p70 (r = 0.28, *p* = 0.001), MCP-1 (r = 0.32, *p* = 0.0002), MCP-3 (r = 0.21, *p* = 0.02), RANTES (r = 0.30, *p* = 0.0005), TNF-α (r = 0.29, *p* = 0.0008), and TAR L2: IL-1β (r = 0.24, *p* = 0.006), IL-10 (r = −0.24, *p* = 0.006), IL-12 p70 (r = 0.24, *p* = 0.006), IL-16 (r = 0.20, *p* = 0.02), MCP-1 (r = 0.28, *p* = 0.001), MCP-3 (r = 0.19, *p* = 0.03), MIF (r = −0.20, *p* = 0.02), RANTES (r = 0.21, *p* = 0.02), SCF (r = 0.22, *p* = 0.01), and TNF-α (r = 0.22, *p* = 0.01).

There were negative correlations between TBR L1 and IL-1β (r = −0.29, *p* = 0.0008), IL-3 (r = −0.23, *p* = 0.008), IL-4 (r = −0.24, *p* = 0.006), IL-16 (r = −0.23, *p* = 0.008), IL-18 (r = −0.20, *p* = 0.02), LIF (r = −0.21, *p* = 0.02), MCP-3 (r = −0.34, *p* < 0.0001), MIP-1α (r = −0.23, *p* = 0.008), β-NGF (r = −0.26, *p* = 0.004), and TNF-α (r = −0.23, *p* = 0.008). Additionally, we found negative correlations between TBR L2 and IL-1Ra (r = −0.21, *p* = 0.02), IL-3 (r = −0.22, *p* = 0.01), IL-16 (r = −0.20, *p* = 0.02), MCP-3 (r = −0.22, *p* = 0.01), and G-CSF (r = −0.23, *p* = 0.008), and a positive correlation between TBR L2 and PDGF-BB (r = 0.23, *p* = 0.008).

### 3.4. Serum Concentrations of Cytokines and Growth Factors in Subjects with T1D: Relationships with GV

We compared the concentrations of the studied cytokines and growth factors in patients with CV values < 36% and in those with CV ≥ 36%. Most of the molecules demonstrated no significant differences between the groups (Table 4). However, MCP-1 and RANTES were markedly higher and the MIF level was decreased in patients with unstable glucose levels. Both diabetic groups showed significant differences with the control group in concentrations of IL-1α, IL-1β, IL-2Rα, IL-3, IL-4, IL-6, IL-7, IL-16, IL-17A, LIF, GM-CSF, M-CSF, IFN-γ, MCP-1, MCP-3, and β-NGF.

In subjects with T1D, CV correlated positively with concentrations of MCP-1 (r = 0.18, *p* = 0.04) and RANTES (r = 0.27, *p* = 0.002). There were negative correlations between CV and IL-10 (r = −0.23, *p* = 0.008) and MIF (r = −0.26, *p* = 0.003). MAGE demonstrated positive correlations with IL-1β (r = 0.19, *p* = 0.03), IL-12 p70 (r = 0.29, *p* = 0.0008), MCP-1 (r = 0.21, *p* = 0.02), RANTES (r = 0.27, *p* = 0.02), and SDF-1α (r = 0.21, *p* = 0.02). There were negative correlations between MAGE and IL-8 (r = −0.19, *p* = 0.03), IL-10 (r = −0.22, *p* = 0.01), and MIF (r = −0.25, *p* = 0.004). MAG showed positive correlations with IL-12 p70 (r = 0.39, *p* < 0.0001), MCP-1 (r = 0.31, *p* = 0.0003), and SDF-1α (r = 0.26, *p* = 0.003).

### 3.5. Serum Concentrations of Cytokines and Growth Factors in Subjects with T1D: Other Relationships

In the panel of studied molecules, IL-1β, MCP-3, and TNF-α only showed weak positive correlations with HbA1c (r = 0.23, *p* = 0.008; r = 0.19, *p* = 0.03; r = 0.21, *p* = 0.02, respectively). Additionally, IL-1β, IL-2Rα, and IL-16 demonstrated negative correlations with C-peptide (r = −0.25, *p* = 0.004; r = −0.19, *p* = 0.03; r = −0.26, *p* = 0.003, respectively). None of the cytokines were associated with age.

Patients with BMI ≥ 25 kg/m^2^ when compared to those with BMI < 25 kg/m^2^ demonstrated higher levels of IL-1β (3.9, 3.0–4.4 vs. 3.3, 2.5–3.9 pg/mL, *p* = 0.003), IL-12 p70 (7.0, 3.9–13.7 vs. 4.1, 2.7–5.7 pg/mL, *p* = 0.005), IP-10 (257, 7.5–490 vs. 88, 4.5–372 pg/mL, *p* = 0.03), LIF (26.5, 8.6–59.3 vs. 8.6, 0–49.4 pg/mL, *p* = 0.006), MCP-3 (6.0, 0–6.8 vs. 1.0, 0–6.2 pg/mL, *p* = 0.02), β-NGF (0, 0–1.9 vs. 1.4, 0–4.7 pg/mL, *p* = 0.002), and TNF-α (7.4, 3.0–75 vs. 4.8, 0.56–54 pg/mL, *p* = 0.02) and lower levels of IL-10 (4.3, 2.9–5.3 vs. 5.2, 3.8–6.0 pg/mL, *p* = 0.049). Other cytokines did not demonstrate statistically significant differences (data are not shown). BMI correlated positively with IL-1β (r = 0.20, *p* = 0.02), IL-16 (r = 0.23, *p* = 0.008), IP-10 (r = 0.20, *p* = 0.02), LIF (r = 0.19, *p* = 0.03), MCP-3 (r = 0.20, *p* = 0.02), and β-NGF (r = 0.21, *p* = 0.02). There was a negative correlation between BMI and IL-10 (r = −0.20, *p* = 0.02).

### 3.6. Multiple Regression Models

In logistic regression models, the levels of IL-1β, IL-4, IL-10, IL-12 (p70), GM-CSF, HGF, MCP-3, MIF, and TNF-α were associated with TIR ≤ 70% (Table 5). All these molecules, excluding MIF, demonstrated significant associations with TIR ≤ 70% after adjustments for age, sex, BMI, diabetes duration, and eGFR. The concentrations of MIF and PDGF-BB were associated with CV ≥ 36%.

## 4. Discussion

In this study, we assessed serum levels of 44 cytokines and growth factors that are discussed as mediators of diabetic complications in subjects with T1D depending on CGM-derived TIRs and GV metrics. To determine the circulating regulators, we used multiplex analysis, or multiplex bead array assay. This method makes it possible to simultaneously determine a large number of biomarkers in one biological sample [20,21]. Moreover, we performed a comprehensive analysis of the GV [10,27] that included an assessment of time-dependent parameters (TIR, TAR L1, TAR L2, TBR L1, TBR L2), the dispersion of glucose values (CV), the amplitude of glucose fluctuations (MAGE), and the rate of glucose changes (MAG).

The results demonstrate significant changes in the levels of the studied regulators in subjects with T1D as compared with normoglycemic individuals. In particular, we recorded an increase in the levels of pro-inflammatory cytokines (IL-1β, IL-6, IL-12 p70, IL-16, IL-17A, IFN-γ, TNF-α), monocyte chemoattractant proteins (MCP-1, MCP-3), a depletion of an anti-inflammatory cytokine (IL-4), an imbalance among hematopoietic growth factors (IL-3, IL-7, M-CSF, GM-CSF), and changes in the concentrations of other regulators of the immune system and cell differentiation (IL-1α, IL-1Ra, IL-2Rα, IFN-α2, LIF). In general, these findings are consistent with the literature data on an increase in the levels of circulating IL-1β [28,29,30], IL-6 [29,31,32], IL-12 [29], IL-17A [30,33], IFN-γ [32,34], MCP-1 [29,35], TNF-α [28,29,32,36], and a decrease in IL-4 concentrations [37] in patients with T1D. However, decreased concentrations of IL-6 [28] and MCP-1 [38,39] in T1D patients were also reported. Other authors demonstrated no differences between healthy individuals and people with diabetes in serum IL-4 [40] and MCP-1 [41]. The discrepancies in the data can be explained by the heterogeneity of the included patients in terms of age, diabetes duration, the presence of complications, and the quality of glycemic control. 

When interpreting the levels of cytokines in subjects with T1D, the autoimmune nature of the disease should be taken into account. Some studies have matched the levels of circulating cytokines with markers of the autoimmune process in patients with T1D. It was reported that children with one or two diabetes-related antibodies (IA-2 and/or GAD65) have significantly higher levels of IL-1β and IL-2 and lower levels of IL-4 than children with diabetes who were negative for these markers [40]. In patients with recent-onset T1D, an increase in IL-18 and decrease in MIF and MCP-1 levels were associated with IA-2 and GAD65 antibody positivity [42]. Another study showed a dependency of accelerated autoimmunity and beta cell destruction on increased IFN-γ, IL-12, and IL-17 and decreased IL-4, IL-6, and IL-13 in pediatric patients with T1D [37]. The onset of T1D in children was characterized by the upregulation of GM-CSF, IL-1β, IL-7, IL-8, IL-10, IL-17F, IL-21, IL-23, and IL-27, but not IL-6 or TNF-α; the presence of autoantibodies (anti-IA-2 and -ZnT8) influenced the blood cytokine levels [43]. In this study, we did not test patients for autoimmune markers of diabetes but assessed fasting C-peptide levels. Though we found negative correlations between fasting C-peptide and IL-1β, IL-2Rα, and IL-16 concentrations, it should be emphasized that the majority of patients in our study had long-term diabetes with undetectable beta-cell function. Therefore, the autoimmune process hardly played a significant role in changing the cytokine profile in our cohort.

The greatest changes in the levels of the studied molecules were revealed in patients with TIR values < 70%. Compared to individuals with target TIR, these patients showed higher concentrations of IL-1β, IL-1Ra, IL-2Rα, IL-3, IL-6, IL-7, IL-12 p70, IL-16, IL-17A, G-CSF, M-CSF, IFN-γ, MCP-1, MCP-3, TNF-α, TNF-β, SCF, and HGF, and lower levels of IL-1α, IL-4, IL-8, IL-10, GM-CSF, and MIF. Associations of IL-1β, IL-4, IL-10, IL-12 (p70), GM-CSF, HGF, MCP-3, and TNF-α with target TIR were confirmed in a multivariate logistic analysis. It is interesting to note that many of the studied molecules showed associations with TIR, but not with HbA1c. Naturally, TIR reflects glucose levels over a much shorter period than HbA1c. Therefore, it can be hypothesized that recent glucose fluctuations may have a greater impact on cytokine production than long-term glycemic control. Our data on the associations of TIR with the levels of important biological regulators represent another argument for the widespread use of TIR in assessing glycemic control in people with diabetes.

Most of the TIR-associated molecules also showed correlations with TAR L1 and TAR L2. This is consistent with the literature data on the pro-inflammatory effect of hyperglycemia in diabetes. In vitro experiments showed a stimulating effect of high glucose on the production of principal proinflammatory cytokines in cultured human mononuclear cells [44,45,46], B cells [47], human umbilical vein endothelial cells [48], aortic endothelial cells [49], glomerular endothelial cells [50], cardiomyocytes [51], and beta cells [52]. According to these data, a high glucose level increases the secretion of IL-1β [46,50,52], IL-6 [46,51], IL-12 [44], IFN-γ [46], TNF-α [45,46,49,51], MCP-1 [48,49], and other proinflammatory cytokines. These findings are consistent with our results. Moreover, we found a depletion in the levels of anti-inflammatory cytokines (IL-4, IL-10) in patients with non-target TIR, which confirmed the imbalance between inflammatory and anti-inflammatory cytokines under hyperglycemic conditions.

In our study, many molecules were associated with time-dependent parameters reflecting hyperglycemic fluctuations (TAR L1 and TAR L2), and some molecules (IL-1β, IL-8, IL-12 p70, MCP-1, RANTES, MIF, SDF-1α) demonstrated correlation(s) with at least one amplitude-dependent GV metric (CV, MAGE, and/or MAG indices). It has previously been shown that intermittently high glucose enhances the secretion of IL-6 and TNF-α by activated monocytes [53]. Moreover, it has been demonstrated that glucose oscillations stimulate IL-6 production in endothelial cells to a greater extent than persistently high glucose [54,55]. Similarly, acute glucose fluctuations were a more potent inducer of IL-1β and TNF-α in rat podocytes than constantly high glucose [56]. In adipocytes, oscillating glucose induced a greater increase in MCP-1 secretion than constantly high glucose [57]. In rats, blood glucose fluctuations induced by intermittent glucose infusions increased the expression of IL-6 and TNF-α in vascular endothelial cells [58]. Therefore, the effect of hyperglycemia on cytokine synthesis may be exacerbated by that of GV.

Hypoglycemia may act as an additional trigger of inflammation in individuals with high GV. In cultured macrophages, intermittent episodes of hypoglycemia and hyperglycemia promote M1 polarization and enhance IL-1β, TNF-α, IL-6, and MCP-1 secretion [59]. In healthy individuals and those with T1D, hypoglycemia promotes leukocyte mobilization into the bloodstream and induces proinflammatory changes in these cells [60]. In individuals with T1D, an episode of two-hour hypoglycemia was followed by an increase in the levels of IL-6 [61]. High blood glucose, replacing hypoglycemia, caused a further increase in the concentrations of IL-6 [62]. Contrary to these data, we found weak negative correlations between TBR and some studied cytokines. This can be explained by associations of these molecules with TIR and TAR, which are in reciprocal relationships in TBR. It should also be noted that all hypoglycemic episodes in our study were non-severe and may not have affected cytokine levels.

When discussing possible confounders affecting cytokine levels in patients with T1D, BMI should be taken into account [63]. The prevalence of obesity among people with T1D is increasing [64] and nearly a quarter of T1D patients are affected by metabolic syndrome [65]. In our study, patients with excessive BMI had higher levels of IL-1β, IL-12 p70, IP-10, LIF, MCP-3, β-NGF, and TNF-α, and lower levels of IL-10. However, associations of IL-1β, IL-10, IL-12 p70, MCP-3, and TNF-α with TIR remained significant after adjustment to BMI, as well as other possible confounders (age, sex, diabetes duration, and eGFR). This demonstrates an independent association of excessive glucose oscillations with the changes in cytokine levels.

Our study is not without limitations. The recruitment of patients in one center could lead to some bias in the sample selection. A cross-sectional design does not prove the causality. The CGM duration was rather short. However, to the best of our knowledge, this is the first study that examined a panel of circulating cytokines and growth factors depending on GV parameters in T1D patients. The strengths of the study are a comprehensive analysis of GV with time-dependent and amplitude-dependent metrics and a determination of the relationship of these parameters with the levels of a large number of cytokines and growth factors. As a result, we have identified the regulators most associated with off-target glucose values and GV. Further studies are needed to explore the effect of glucose fluctuations on their production and signaling. At the same time, identified molecules can be considered to be perspective biomarkers of GV-related immune dysfunction in T1D.

## 5. Conclusions

Subjects with T1D demonstrated an increase in serum concentrations of IL-1β, IL-1Ra, IL-2Rα, IL-3, IL-6, IL-7, IL-12 p40, IL-16, IL-17A, LIF, M-CSF, IFN-α2, IFN-γ, MCP-1, MCP-3, and TNF-α compared to normoglycemic individuals. The changes in the levels of these cytokines and growth factors are related to the time-dependent and amplitude-dependent GV characteristics. Specifically, the levels of IL-1β, IL-10, IL-12 p70, IL-16, MCP-1, MCP-3, RANTES, TNF-α, SCF, and MIF are associated with TAR, while IL-1β, IL-8, IL-12 p70, MCP-1, RANTES, MIF, and SDF-1α show association with at least one amplitude-dependent GV parameter (CV, MAGE, and MAG). Therefore, the pro-inflammatory effect of hyperglycemia and GV in diabetes may be realized through the shifts in cytokine and chemokine production. The study results provide further support for the notion that GV may be a therapeutic target in the management of diabetes.

## Figures and Tables

**Table 1:** Clinical characteristics of the study participants with T1D.

Parameter	Median (25; 75 Percentile)
Demographic and general clinical parameters	
Age, years	33 (24; 43)
Smokers, *n* (%)	23 (18%)
BMI, kg/m^2^	23 (20; 26)
Waist-to-hip ratio	0.81 (0.76; 0.91)
Diabetes-related parameters and associated diseases	
Diabetes duration, years	15 (10; 23)
Daily insulin dose, IU/kg	0.7 (0.5; 0.9)
Diabetic retinopathy, *n* (%)	79 (61%)
Chronic kidney disease, *n* (%)	75 (58%)
Diabetic neuropathy, *n* (%)	96 (74%)
Arterial hypertension, *n* (%)	39 (30%)
Coronary artery disease, *n* (%)	7 (5.3%)
Peripheral artery disease, *n* (%)	18 (14%)
Laboratory parameters	
HbA1c, %	7.9 (6.8; 9.6)
HbA1c, mmol/L	67 (51; 81)
Total cholesterol, mmol/L	5.1 (4.2; 6.3)
LDL-cholesterol, mmol/L	3.0 (2.4; 3.7)
HDL-cholesterol, mmol/L	1.5 (1.3; 1.8)
Triglycerides, mmol/L	1.0 (0.7; 1.3)
hsCRP, mmol/L	1.3 (0.7; 2.8)
eGFR (CKD-EPI formula, 2009), mL/min/1.73 m^2^	94 (82; 105)
UACR, mg/mmol	0.5 (0.3; 1.1)
Hemoglobin, g/L	139 (125; 150)
RBC, ×10^12^	4.7 (4.4; 5.0)
WBC, ×10^9^	5.4 (4.8; 6.9)

Continuous data are presented as medians (lower quartile; upper quartile), and frequencies are presented as number of patients (percentage). BMI, body mass index; eGFR, estimated glomerular filtration rate; HbA1c, glycated hemoglobin A1c; HDL, high density lipoprotein; hsCRP, high sensitivity C-reactive protein; LDL, low-density lipoprotein; RBC, red blood cells; UACR, urinary albumin-to-creatinine ratio; WBC, white blood cells.

**Table 2:** CGM-derived TIRs and GV parameters in subjects with T1D.

Parameter	Median (25; 75 Percentile)
Mean glucose, mmol/L	7.7 (6.6; 9.3)
TIR, %	72 (57; 88)
TAR L-1, %	17 (7.4; 25)
TAR L-2, %	3.5 (0.3; 11)
TBR L-1, %	1.5 (0; 2.1)
TBR L-2, %	0.2 (0; 1.2)
CV, %	33 (27; 38)
MAGE, mmol/L	4.3 (3.1; 5.2)
MAG, mmol × h^−1^ × L^−1^	1.7 (2.1; 2.4)

Data are presented as median (lower quartile; upper quartile). TIR, time in range; TAR L-1, time above range, level 1; TAR L-2, time above range, level 2; TBR L-1, time below range, level 1; TBR L-2, time below range, level 2; CV, coefficient of variation; MAGE, mean amplitude of glycemic excursions; MAG, mean absolute glucose change.

**Table 3:** Serum concentrations (pg/mL) of cytokines and growth factors in T1D subjects depending on TIR.

Molecule	Group		*p*
	TIR > 70% (*n* = 69)	TIR ≤ 70% (*n* = 61)	
IL-1α	0.93 (0; 6.42) **	0 (0; 3.82) ***	**0.01**
IL-1β	3.07 (2.53; 3.90) ***	3.79 (3.06; 4.31) ***	**0.0004**
IL-1Ra	296 (4.46; 461)	405 (7.25; 478) **	0.08
IL-2	7.94 (3.42; 9.55)	6.12 (1.07; 9.56)	0.09
IL-2Rα	18 (3.24; 63) ***	30 (4.96; 54) ***	0.68
IL-3	0.53 (0.45; 0.65) ***	0.56 (0.49; 0.70)***	0.07
IL-4	3.82 (1.10; 5.88) ***	1.53 (0.42; 3.95) ***	**0.01**
IL-5	42 (5.14; 77)	9.89 (4.14; 117)	0.96
IL-6	4.32 (2.40; 5.86)	5.54 (3.84; 7.03) ***	**0.007**
IL-7	10 (3.91; 18) **	11 (3.78; 18) ***	0.78
IL-8	6.53 (2.28; 9.38)	3.83 (1.52; 6.90) *	0.07
IL-9	182 (5.22; 251)	6.64 (2.92; 235)	0.07
IL-10	5.29 (3.83; 6.05)	4.75 (2.90; 5.29) *	**0.001**
IL-12 p40	3.62 (0; 9.66)	0.28 (0; 9.42)	0.25
IL-12 p70	4.10 (3.41; 5.41)	6.56 (3.41; 14) **	**0.02**
IL-16	9.12 (3.74; 36) *	34 (6.08; 490) ***	**0.02**
IL-17A	10 (4.46; 15) *	8.95 (6.09; 13) **	0.94
IL-18	8.34 (3.29; 23)	8.01 (4.02; 32)	0.35
LIF	8.58 (0; 49) ***	26 (8.58; 59)	**0.02**
G-CSF	14 (2.46; 72) **	38 (5.48; 74) *	0.16
GM-CSF	4.66 (0.83; 6.04)	1.08 (0; 5.08) ***	**0.002**
M-CSF	9.29 (3.67; 18)	12 (4.4; 21) ***	0.25
GRO-α	0 (0; 5.46)	0 (0; 0.75)	0.2
IFN-α2	0 (0; 1.62) *	0 (0; 1.62)	0.71
IFN-γ	1.79 (1.09; 3.06) ***	1.94 (1.07; 2.53) ***	0.87
IP-10	145 (4.57; 333)	235 (6.09; 471)	0.13
MCP-1	6.83 (3.11; 30) *	24.39 (6.48; 41) ***	**0.01**
MCP-3	0 (0; 5.70) **	6.05 (0.91; 6.74) ***	**0.0006**
MIF	8.44 (0; 149)	0.44 (0; 28) **	**0.005**
MIG	110 (6.23; 177)	117 (5.95; 200)	0.84
MIP-1α	1.54 (1.18; 2.22)	1.81 (1.50; 2.58)	0.07
MIP-1β	193 (4.51; 224)	7.98 (4.07; 232)	0.61
RANTES	2437 (3.14; 11,840)	9590 (6.99; 14,106)	**0.02**
TNF-α	4.25 (0; 12)	6.77 (3.29; 79) ***	**0.002**
TNF-β	176 (4.30; 226)	208 (6.72; 289) *	**0.04**
TRAIL	7.47 (5.35; 13)	8.49 (3.79; 13)	0.95
SCF	19 (3.10; 78)	69 (6.48; 99) *	0.06
SCGF-β	9.01 (4.57; 117,138)	9.85 (3.87; 126,518)	0.92
SDF-1α	1276 (4.63; 1611)	1339 (1132; 1543)	0.26
bFGF	20 (3.82; 28)	8.33 (7.06; 108)	0.75
PDGF-BB	385 (5.66; 1307)	730 (4.78; 1402)	0.58
HGF	159 (5.42; 326)	291 (6.28; 407) *	0.06
β-NGF	0 (0; 1.24) *	1.24 (0; 4.69)	**0.02**
VEGF	152 (6.11; 208)	6.11 (2.87; 214)	0.22

Data are presented as medians (lower quartile; upper quartile). * *p* < 0.05, ** *p* < 0.01, *** *p* < 0.001 vs. subjects with NGT. Significant differences between diabetic groups are highlighted in bold. NGT, normal glucose tolerance; T1D, type 1 diabetes; TIR, time in range.

**Table 4:** Serum concentrations (pg/mL) of cytokines and growth factors in T1D subjects depending on CV.

Molecule	Group		*p*
	CV < 36% (*n* = 72)	CV ≥ 36% (*n* = 58)	
IL-1α	0.46 (0; 4.85) ***	0.51 (0; 4.05) ***	0.74
IL-1β	3.60 (2.62; 4.10) ***	3.48 (2.71; 4.10) ***	0.94
IL-1Ra	324 (5.81; 464)	376 (5.81; 478) *	0.69
IL-2	7.78 (2.35; 9.93)	7.20 (1.07; 9.24)	0.16
IL-2Rα	21 (4.96; 59) ***	25 (4.96; 54) ***	0.99
IL-3	0.57 (0.46; 0.69) ***	0.53 (0.49; 0.63) ***	0.23
IL-4	4.89 (2.40; 6.26) ***	5.17 (3.33; 6.69) ***	0.33
IL-5	39 (5.03; 98)	38 (4.63; 90)	0.40
IL-6	3.08 (0.90; 4.86) **	2.73 (0.17; 5.29) **	0.76
IL-7	11 (4.03; 18) ***	10 (3.78; 18) **	0.63
IL-8	5.69 (2.43; 9.26)	4.41 (1.52; 8.10)	0.15
IL-9	7.85 (3.71; 244)	11 (4.35; 258)	0.55
IL-10	4.96 (3.83; 5.66)	4.75 (2.90; 5.66)	0.45
IL-12 p40	2.92 (0; 14)	0.38 (0; 6.09) *	0.33
IL-12 p70	4.33 (3.41; 13)	4.45 (3.41; 13)	0.73
IL-16	31 (5.74; 45) **	9.12 (4.87; 38) *	0.26
IL-17A	9.15 (4.85; 15) *	9.36 (5.77; 15) **	0.57
IL-18	8.85 (3.86; 24)	7.37 (3.89; 25)	0.98
LIF	8.58 (0.43; 49) ***	22 (4.92; 54) ***	0.29
G-CSF	38 (3.30; 87)	22 (3.37; 60)	0.53
GM-CSF	2.85 (0.24; 5.40) ***	2.94 (0; 6.28) *	0.96
M-CSF	9.78 (3.89; 18) **	9.66 (3.89; 20) **	0.77
GRO-α	0 (0; 4.34)	0 (0; 0.75)	0.45
IFN-α2	0 (0; 1.62)	0 (0; 3.35) *	0.27
IFN-γ	1.87 (1.08; 2.98) ***	1.94 (1.07; 2.48) ***	0.67
IP-10	180 (5.62; 442)	193 (6.09; 448)	0.87
MCP-1	6.91 (3.18; 30) *	24 (6.75; 43) ***	**0.007**
MCP-3	5.34 (0; 6.63) ***	4.74 (0; 6.28) ***	0.72
MIF	14 (0; 144)	1.59 (0; 20) **	**0.04**
MIG	115 (6.23; 217)	112 (6.11; 193)	0.79
MIP-1α	1.63 (1.35; 3.06)	1.74 (1.41; 2.24)	0.65
MIP-1β	173 (3.95; 224)	96 (4.99; 230)	0.58
RANTES	9.85 (4.07; 12,458)	9868 (7.22; 13,970)	**0.03**
TNF-α	5.21 (0.67; 62)	6.53 (2.77; 60) *	0.61
TNF-β	170 (4.77; 236)	208 (6.96; 266) *	0.18
TRAIL	7.78 (5.12; 12)	7.98 (3.79; 13)	0.93
SCF	14 (3.65; 88)	69 (6.68; 99) *	0.18
SCGF-β	10,314 (4.04; 115,521)	8.39 (4.65; 130,694)	0.64
SDF-1α	1298 (6.48; 1580)	1314 (8.44; 1569)	0.55
bFGF	20 (5.26; 34)	8.33 (5.64; 28)	0.36
PDGF-BB	190 (3.80; 1212)	794 (5.38; 1544)	0.11
HGF	219 (5.33; 328)	288 (6.72; 403) *	0.19
β-NGF	0.00 (0; 4.07) **	0 (0; 3.18) **	0.53
VEGF	167 (3.39; 226)	6.18 (3.31; 198)	0.21

Data are presented as medians (lower quartile; upper quartile). * *p* < 0.05, ** *p* < 0.01, *** *p* < 0.001 vs. subjects with NGT. Significant differences between diabetic groups are highlighted in bold. CV, coefficient of variation; NGT, normal glucose tolerance; T1D, type 1 diabetes.

**Table 5:** Serum cytokines and growth factors associated with TIR ≤ 70% and CV ≥ 36% in subjects with T1D.

Molecule	Crude OR (95% CI), *p*-Value	Adjusted OR (95% CI), *p*-Value
TIR ≤ 70%		
IL-1β, 1 pg/mL	1.78 (1.18–2.67), *p* = 0.006	1.69 (1.12–2.55), *p* = 0.01
IL-4, 1 pg/mL	0.82 (0.71–0.95), *p* = 0.007	0.82 (0.7–0.96), *p* = 0.01
IL-10, 1 pg/mL	0.7 (0.56–0.89), *p* = 0.003	0.73 (0.56–0.94), *p* = 0.01
IL-12 (p70), 1 pg/mL	1.08 (1.02–1.15), *p* = 0.006	1.08 (1.01–1.15), *p* = 0.02
MCP-3, 1 pg/mL	1.16 (1.04–1.29), *p* = 0.006	1.14 (1.02–1.28), *p* = 0.03
MIF, 100 pg/mL	0.76 (0.6–0.97), *p* = 0.03	0.79 (0.62–1.01), *p* = 0.06
TNF-α, 10 pg/mL	1.14 (1.03–1.27), *p* = 0.01	1.12 (1–1.25), *p* = 0.04
GM-CSF, 1 pg/mL	0.82 (0.72–0.94), *p* = 0.005	0.84 (0.73–0.97), *p* = 0.02
HGF, 100 pg/mL	1.21 (1.02–1.44), *p* = 0.03	1.23 (1.02–1.47), *p* = 0.03
CV ≥ 36%		
MIF, 100 pg/mL	0.78 (0.62–0.99), *p* = 0.04	0.78 (0.61–0.99), *p* = 0.04
PDGF-BB, 1000 pg/mL	1.56 (1.05–2.32), *p* = 0.03	1.58 (1.05–2.37), *p* = 0.03

Logistic regression models. Adjustment to age, sex, BMI, diabetes duration, and eGFR. CV, coefficient of variation; T1D, type 1 diabetes; TIR, time in range.

## Data Availability

The data that support the findings of this study are available from the corresponding author [V.V.K.] upon reasonable request.
